# *Mycobacterium tuberculosis* biology, pathogenicity, and interaction with the host

**DOI:** 10.1038/s41579-025-01201-x

**Published:** 2025-06-30

**Authors:** Digby F. Warner, Amy K. Barczak, Maximiliano G. Gutierrez, Valerie Mizrahi

**Affiliations:** 1 Molecular Mycobacteriology Research Unit, Institute of Infectious Disease and Molecular Medicine and Department of Pathology, University of Cape Town, Cape Town, South Africa; 2 Discovery Research Platform for Infection, Centre for Infectious Diseases Research in Africa, University of Cape Town, Cape Town, South Africa; 3 Division of Infectious Diseases, Massachusetts General Hospital, Boston, Massachusetts, USA; 4 Ragon Institute of Mass General, MIT and Harvard, Boston, Massachusetts, USA; 5 Department of Medicine, Harvard Medical School, Boston, Massachusetts, USA; 6 The Francis Crick Institute, London, UK

## Abstract

Since the release of the first *Mycobacterium tuberculosis* genome in 1998, major advances have been made in understanding the biology of this pathogen, the leading infectious cause of death in modern human history. In this Review, we outline the physiological and metabolic features thought to underpin the survival, evasion, and subversion strategies employed by *M. tuberculosis* as it drives a cycle of transmission, infection, and disease in its obligate human host. We also consider adaptations to key host innate immune effectors, including the roles of granulocytes, phagosomal damage and repair, autophagy, and cell death in determining host–mycobacterium outcomes. Given increasing awareness of the importance of asymptomatic *M. tuberculosis* infection and transmission, we advocate for the need to ensure greater intersection between laboratory and clinical research, taking into account the environmental context in which natural infection and disease occur. We identify knowledge gaps in the field and reflect on the opportunities and challenges for integrating host, bacterium, and environment into future investigations to inform intervention strategies to control tuberculosis disease.

## Introduction

In 2023, *Mycobacterium tuberculosis*, the leading infectious killer of humankind^1^, caused 10.8 million new cases of tuberculosis (TB) and 1.25 million deaths globally^2^. Though regularly cited among archetypal bacterial pathogens, full understanding of *M. tuberculosis* requires a deeper insight into the adaptation of the bacillus to its obligate human host and the appreciation that *M. tuberculosis* infection (possibly transmission, too^3^) is asymptomatic in most individuals, even in high TB-burden regions^4^. The absence of symptoms despite demonstrable bacillary infection — for example, in culture-positive individuals sampled in both clinic and community settings^5^ — represents a major shift from traditional thinking, which has assumed an inexorable link between *M. tuberculosis* infection and TB disease^6^. Put simply, long-term *M. tuberculosis* survival — and, potentially, circulation — within human populations is a composite outcome, with mycobacterial virulence a contributing, but not deciding, trait. To ascertain the risks of disease development, a comprehensive understanding of the host–mycobacterium interaction is needed, and must include the factors leading to benign outcomes in the majority of individuals exposed to *M. tuberculosis*^7^. This is currently lacking and, combined with the skewed prevalence of TB towards low-income settings (more than 85% of incident disease occurs in 30 high-burden countries)^2^, reinforces the importance of the interplay between mycobacterial, host, and environmental factors in sustaining elevated TB rates in endemic regions^8^. Recognizing this interplay argues for greater integration of all three components (mycobacterium, host, environment) in TB research, primarily by refocusing on humans as natural host^7^. This is challenging to accomplish, given that major advances have derived from in vitro, ex vivo, and animal models of infection, which only partially incorporate these different determinants^9^. However, the use of post-mortem sampling to demonstrate the capacity of *M. tuberculosis* for intrahost genomic diversification and dissemination^10^, the validation of findings from microanatomic analyses of resected human tissue in mouse knockout mutants^11^, and the combination of clinical and genomic data to elucidate determinants of extrapulmonary disease^12^, provide important examples of how combining laboratory and clinical tools and observations can reveal new insights into *M. tuberculosis* biology and TB etiology.

Despite advances in new TB drug^13^ and vaccine^14^ development, and in understanding the spectrum of TB disease^15^, meaningful impact on morbidity and mortality rates remains aspirational^16^. This owes primarily to TB’s complexity^9^, which arises from heterogeneity at the level of the bacillus^17^, the host^18^, and the environment^19^, and is exacerbated by their intertwined evolutionary histories^20^. Mycobacterial heterogeneity has two components: genetic variation, which underlies the classification of *M. tuberculosis* according to phylogenetic lineages^21^ and can influence infection outcomes^22,23^, drug susceptibility^24^ (for example, lineage 1 is associated with reduced susceptibility to pretomanid)^25^ and drug resistance^21^; and phenotypic heterogeneity, by which subpopulations of genetically identical bacilli adapt differently to dynamic environments^26^. An obligate aerobe, *M. tuberculosis* can maintain bioenergetic homeostasis under hypoxic conditions^27^. And, consistent with its autarkic lifestyle^28^, *M. tuberculosis* is a prototroph, possessing a flexible metabolic repertoire and a unique cell membrane^29^ that allows survival within diverse microenvironments during host infection^30^. This combination of structural durability and metabolic flexibility appears central to the success of *M. tuberculosis* as a human pathogen. However, except for anti-TB chemotherapy (drug-resistant (DR)-TB accounts for a quarter of all deaths from antimicrobial resistance^31^), identifying the selective forces that have shaped, and continue to influence, the co-evolution of the bacillus and its obligate host is mostly speculative^32^. Heterogeneity within and between hosts^33^ adds further variability, with genetics, co-infections, and other co-morbidities, nutritional status^34^, and socioeconomic circumstance impacting disease risk^35^, clinical presentation, and pathology. In this Review, we attempt to synthesize recent advances in understanding *M. tuberculosis* as an obligate human pathogen, identify key knowledge gaps, and propose some directions for future research.

## *Mycobacterium tuberculosis* as an obligate human pathogen

### The life cycle of *Mycobacterium tuberculosis*

**Fig. 1:**
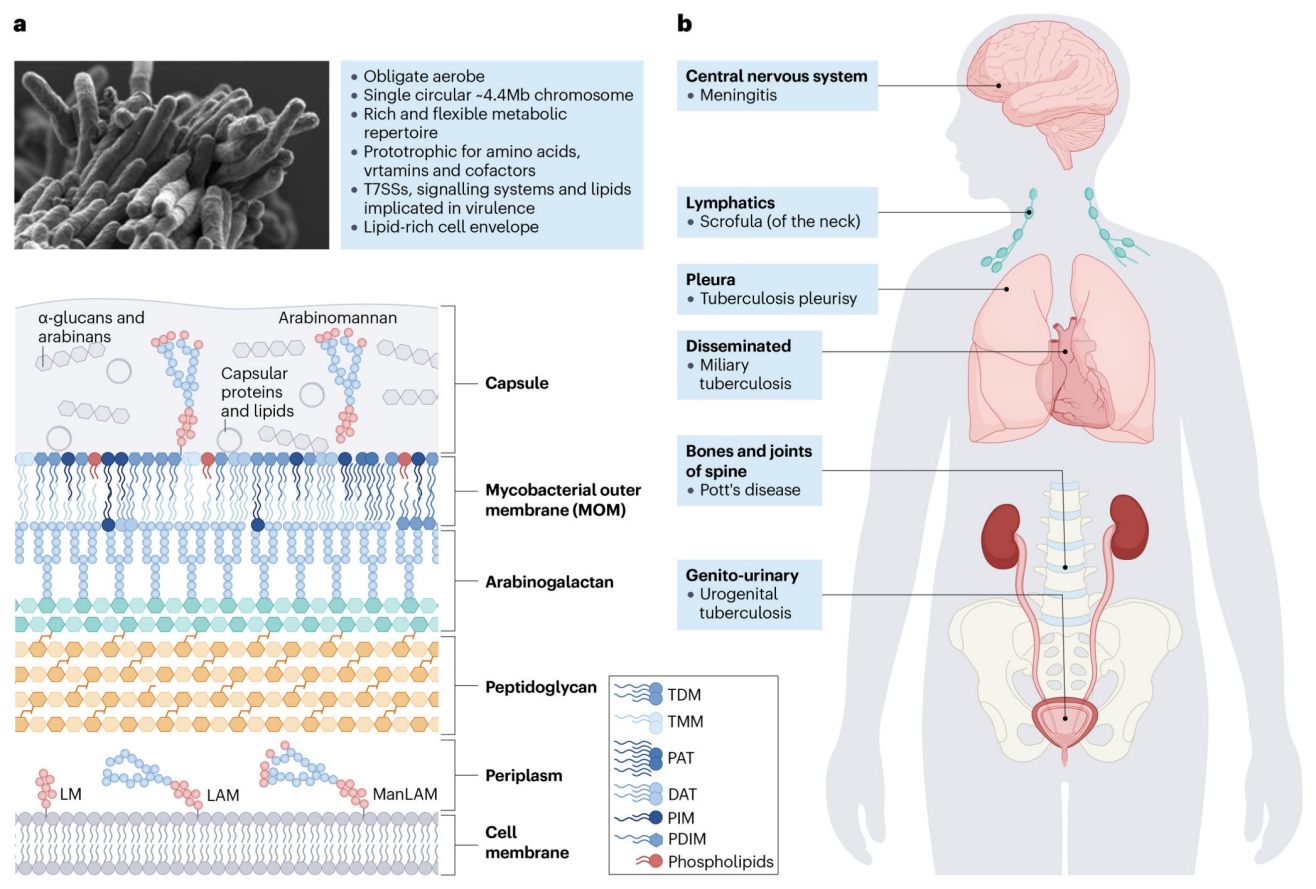
The structure, physiological characteristics and body sites of *Mycobacterium tuberculosis*. a) The tubercle bacillus is a slow-growing, rod-shaped, obligate aerobic bacterium whose key physiological features are summarized in the top figure and described in greater detail in the main text. Shown beneath is a schematic representation of the lipid-rich cell envelope of *M. tuberculosis*. A dynamic, three-dimensional organelle, the cell envelope comprises three main layers: the cell membrane, the cell wall core, and an outer capsule. The core is a highly complex scaffold^187^, containing a crosslinked peptidoglycan polymer that is covalently linked to a highly branched arabinogalactan polysaccharide and long-chain mycolic acids, which form the inner leaflet of the mycobacterial outer membrane (MOM)^187^. The waxy layer of mycolic acids confers hydrophobicity and impermeability and, in the trehalose mono- and di- mycolates, contributes to the formation of hallmark ‘cords’ in growing cells^188^, which have been implicated in innate immune suppression, phagocyte death, and the capacity for intercellular growth between epithelial cells^77^. The mycolic acid layer is, in turn, surrounded by a lipid-rich outer membrane^189^ containing trehalose monomycolate (TMM), trehalose dimycolate (TDM) as well as complex lipoglycans and glycolipids including phthiocerol dimycocerosate (PDIM), phosphoglycolipid, lipoarabinomannan (LAM) and diacyltrehalose (DAT), polyacyltrehalose (PAT), and sulfolipid, which modulate the host immune response. A gelatinous capsule composed of polysaccharides (primarily glucan), proteins, and trace lipids forms the outermost layer of the cell, masking cell envelope components and, with PAT and TDM^190^ provides resistance against desiccation during aerosolisation. The cell envelope is a major target for anti-tuberculosis (TB) drugs^187^. T7SS, type VII secretion system; LM, lipomannan; ManLAM, mannose-capped lipoarabinomannan. b) *M. tuberculosis* can reside not only in the lungs but also in other extra-pulmonary sites such as the central nervous system, lymphatic nodes, the pleura, bones and joints of the spine, the genitourinary tract and the circulatory system. In each of these sites, the mycobacterium can cause a variety of diseases as illustrated in the figure. Part a adapted with permission from ref^191^.

*M. tuberculosis* is a member of the *M. tuberculosis* complex (MTBC)^32^ comprising ten human-adapted and nine animal-adapted lineages that share more than 99.9% sequence identity over homologous regions, yet are associated with distinct global distributions and host tropisms^21^. The first complete genome of *M. tuberculosis* sensu stricto H37Rv revealed a single, circular chromosome ~4.4 megabases in size and encoding ~4000 genes^36^. Comparative genomics analyses have subsequently established the absence of horizontal gene transfer in the evolution of modern *M. tuberculosis* strains^32,37^. As a genetic monomorph^38^, *M. tuberculosis* relies exclusively on single nucleotide polymorphisms and chromosomal deletions, insertions, duplications, conversions, and inversions for genomic variation^37,39,40^, including all resistance-conferring mutations to anti-TB drugs^41^. While the first genome annotation elaborated and explained some known *M. tuberculosis* characteristics, it also revealed unexpected features including large gene complements dedicated to lipid metabolism and arrays of PE-PPE proteins with conserved proline-glutamic acid (PE) or proline-proline-glutamic acid (PPE) motifs at their amino termini^42^, toxin-antitoxin modules^43^, and type VII secretion systems (T7SSs)^44^ (Fig. 1a), whose roles in pathogenicity have been subject of extensive investigation^45^. An unresolved question over the past 25 years has been why an organism with a single host would retain expansive — and for some pathways, apparently redundant — metabolic capacity.

**Fig. 2:**
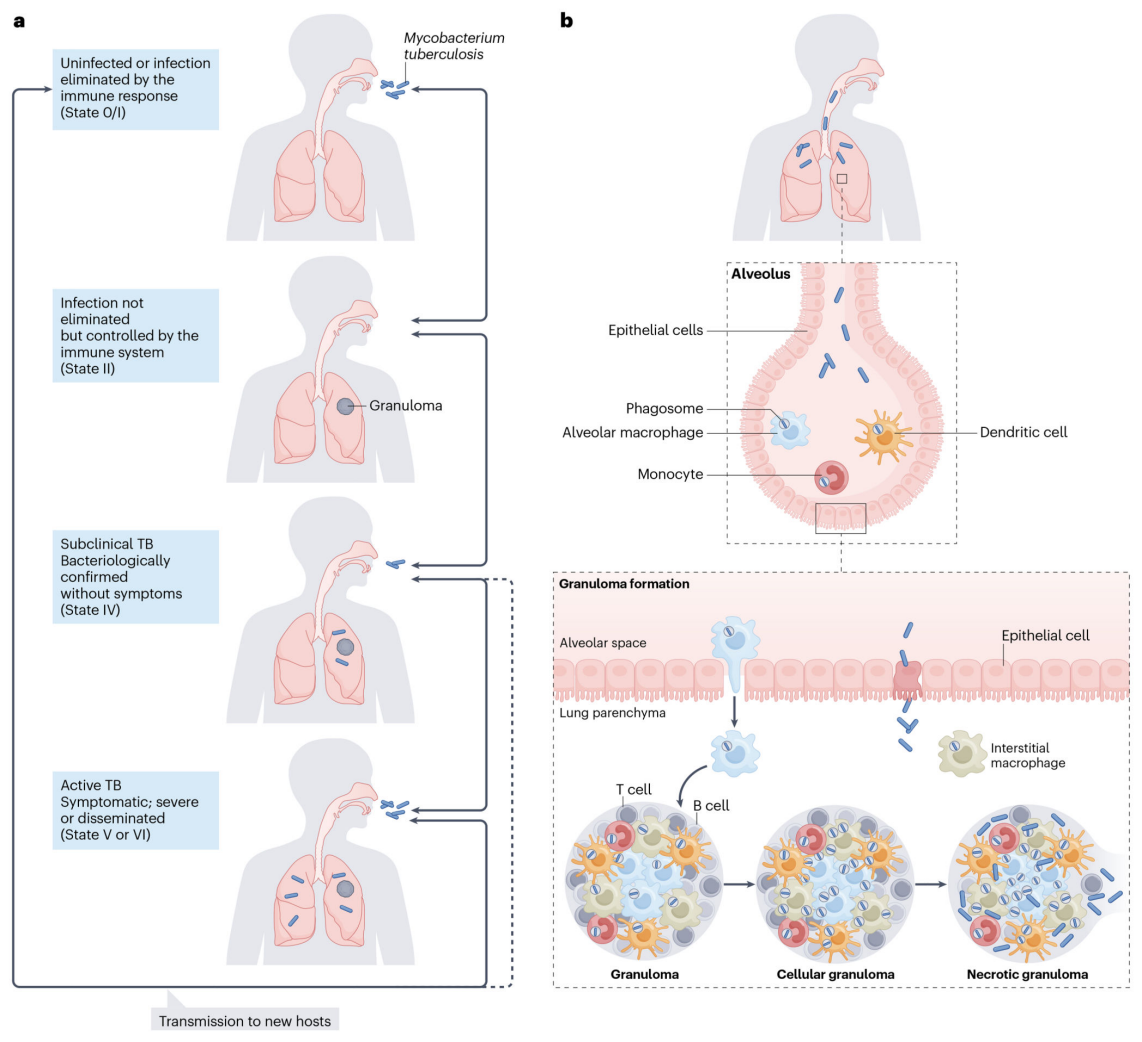
Life cycle and granuloma formation in *Mycobacterium tuberculosis*. a) The cycle of transmission, infection and disease. The cycle starts with the inhalation of aerosolized *M. tuberculosis* by an exposed individual and is followed by a simplified depiction of the spectrum of tuberculosis (TB) disease states^192^. In States 0 and I, the infection has been eliminated by the innate or acquired response, respectively. State 0 also defines individuals with no prior infection, as well as innate resisters who are naturally resistant to acquiring infection. In State II, the infection has not been cleared but is immunologically controlled. In this state, the bacteria reside intracellularly within cellular granulomas. Between States II and IV, albeit not shown in this figure for the sake of simplicity, is State III, in which the infection is no longer controlled by the immune system. This leads to State IV, in which granulomas become necrotic, bacteria are detectable, but the individual is clinically asymptomatic. States V and VI define individuals with active TB, who have signs and symptoms of the disease. The disease severity is high in State VI (for example, cavitary TB; disseminated TB). The cycle is completed by the transmission of aerosolized *M. tuberculosis* to a new host(s). b) *M. tuberculosis* infection leads to granuloma formation^119^. A cellular granuloma can be maintained. Alternatively, bacillary replication occurs resulting in the growth and maturation of the granuloma which begins to necrotize. At this stage, bacilli can be found extracellularly in the necrotic caseum and in immune cells. A lung cavity is formed when an expanding granuloma meets an airway, and its necrotic centre fuses with the structure of the airway. Part b adapted with permission from ref^193^.

*M. tuberculosis* follows a superficially repetitive, albeit erratic^46^ life cycle that is simplistically explained in a series of stages reflecting the most common presentation of TB as pulmonary disease (Fig. 2). For initial infection, airborne *M. tuberculosis* are inhaled deep into lung alveoli where they are engulfed by tissue-resident alveolar macrophages and translocated across the epithelium into the interstitial tissue, initiating infection^47^. Following infection, some viable *M. tuberculosis* cells are transported to the thoracic lymph nodes, where they can proliferate, and drain via the thoracic duct to the bloodstream, facilitating hematogenous spread to any organ, with the apical portions of the lung as a common site of reimplantation^48^. During intracellular survival, the bacteria resist innate immune defences, including macrophage killing, even replicating as the infected macrophages release cytokines that attract more immune cells to form a granuloma, a structured mass that contains the invading bacteria but may also contribute to dissemination^49^. During latent infection, infecting bacilli are contained but not eradicated, and are capable of maintaining a quiescent metabolic state in a clinically asymptomatic host^50^. Upon reactivation (or reinfection), the subversion of immune control allows bacillary replication and antigen release, causing disease. For transmission, coughing^51^ and other respiratory activities^52^ expel aerosol-contained bacilli, reinitiating the infection cycle.

These stages have received the greatest attention since they constitute the ‘productive’ life cycle, in which the entrance and exit of *M. tuberculosis* via the lungs is critical for its onward propagation^53^. However, this introduces the first important nuance in the *M. tuberculosis*–host interaction: the focus in most modern research utilising laboratory and animal models of infection (Box 1) obscures longstanding observations from clinical and pathology studies that differentiate human TB into primary and post-primary disease^7^. In that framing, primary TB (intial infection, intracellular survival and latent infection) centres on the formation of granulomas in a process that establishes systemic immunity, whereas post-primary TB (reactivation or reinfection) is associated with the development of cavities, which facilitate *M. tuberculosis* replication and escape^54^. The concomitant immunity^55^ conferred by primary TB constitutes a double-edged sword, contributing to reactivation of the original infection and progression to post-primary disease in ~5-10% of individuals^54^, and exacerbating the risk of disease from reinfection in settings where the force of infection is high^56^.

The recognition that primary and post-primary TB involve distinct pathological features^7,54^ is critical to understanding *M. tuberculosis* pathogenicity. Implicit in this duality is the notion that intra-host dissemination is essential to all *M. tuberculosis* infections in creating secondary sites of extrapulmonary residence (Fig. 1b), which enable subsequent re-seeding to the lungs to cause post-primary disease^48^. Lymph nodes, especially thoracic, are common sites of infection and may play a crucial role in determining the trajectory of the *M. tuberculosis*–host interaction^57^. This concept is consistent with the presence of the Ghon complex in children and individuals newly infected *M. tuberculosis*, a characteristic lesion of primary TB arising from localized pneumonia at a single infection site, usually in the lower lung^58^. In contrast, post-primary TB manifests in multiple foci in the apical and subapical lobes, possibly reflecting migration of *M. tuberculosis* from infected lymph nodes back to the lungs^48^ or, in TB-endemic settings, reinfection^59^. Notably, work in non-human primates has demonstrated the transport of *M. tuberculosis* from the lungs to the lymph nodes following infection, as well as the implication of lymph nodes in the dissemination of bacilli to the lungs and other organs during reactivation^57^. Though mostly ignored, this ability of *M. tuberculosis* to access other anatomical loci via haematic or lymphatic spread surely requires greater metabolic capacity than is inferred from models of lung infection alone.

A second important nuance in the *M. tuberculosis*–host interaction is that TB must be considered as a spectrum ranging from *M. tuberculosis* infection to clinical disease (Fig. 2), with progression or regression possible at every stage and determined by a poorly understood combination of host, mycobacterial, and environmental factors^15^. Owing to the implications for both population- and patient-level TB control^60^, there has been considerable work towards identifying biomarkers for use as correlates of immune protection^61^ or of risk of disease development^62^; however, while some show promise, further work is required before they can be reliably implemented in real-world settings^63^. A further complication is that asymptomatic TB is increasingly recognized as a potentially major contributor to *M. tuberculosis* transmission^64^. It remains unknown whether the extent of host pathology impacts the physiological state — and, thereby, the infectiousness — of aerosolized bacilli; however, the loss of ‘virulence’ in laboratory-grown organisms suggests this possibility^65^. In this context, the recent demonstration that phylodynamic modelling can be used to infer onset of infectiousness^66^ appears to offer a promising approach.

### The primary niches of *Mycobacterium tuberculosis*

Each new infection initiates with exposure to *M. tuberculosis* released by an infected individual. Though transient, airborne passage of droplet-contained bacilli is essential for *M. tuberculosis* survival, yet is very poorly understood^67^. Resurgent interest in *M. tuberculosis* aerobiology has yielded new insights, including the possibility that functions dispensable for *M. tuberculosis* viability in different infection models might be essential during residence in bioaerosols, for example, as resistance against dessication^68^. In a new advance, an experimental model of transmission was employed to identify the “transmission survival genome” of *M. tuberculosis*^69^. The study revealed a prominent role for proteostasis pathway genes, and identified several conventionally non-essential, transmission-protective genes, including genes potentially involved in dessication resistance^69^. It is also likely that genes identified as highly conserved among diverse *M. tuberculosis* isolates might reflect strong purifying selection on transmission. Comparative analyses extending beyond mycobacterial species indicate that mycolic acids might confer benefits for aerosolization^70,57^.

Following deposition in alveoli, aerosol-borne bacilli are phagocytosed by macrophages, their primary niche^18^. *M. tuberculosis* can survive and replicate inside these cells, thus evading immune elimination while undergoing transport across the epithelium to the lung interstitium. The precise events remain speculative, but observations from animal and ex vivo models^71^ suggest that early proliferation of *M. tuberculosis*-infected macrophages^72^ results in cellular aggregates that recruit monocytes and neutrophils, providing new cellular targets for *M. tuberculosis* dissemination. In addition to alveolar macrophages, there is evidence that *M. tuberculosis* infects microfold cells in the upper respiratory tract^73^, alveolar and bronchial epithelial cells^74^, type II pneumocytes, and fibroblasts. As advanced tools become available, it seems likely that other cell types will be included in the list of possible sites of intracellular occupation; for example, *M. tuberculosis* has recently been detected in CD34^+^ haematopoietic stem cells and ”host-cell accumulated *M. tuberculosis* RNA” cells^74^. There are, moreover, numerous examples of extrapulmonary *M. tuberculosis* infection and disease. In TB endemic regions, as many as 25% of cases are designated as extrapulmonary TB^75^, while the global estimate is around 15% (range 10%-42%)^48^; however, difficulties in diagnosing most extrapulmonary forms means they are severely underreported^75^. Almost any anatomical site can be affected (Fig. 1), and it often involves multiple organ systems^7^. While the implications for onward transmission are assumed to be minimal, the ability to access, and survive in these diverse cellular and anatomical loci reinforces the metabolic adaptability of *M. tuberculosis*. Advances in the manipulation and analysis of human ante-mortem and resected tissue samples^11,74,76^, in combination with appropriate laboratory models^77–80^, provide an encouraging sign that clinical observations can be validated and investigated in tractable in vitro systems, suggesting the potential to iterate models to better reflect in vivo environments.

### The *Mycobacterium tuberculosis* cell envelope: dynamic barrier and host-bacterium interface

Two interlinked concepts appear fundamental to *M. tuberculosis* biology. First, the occupation of humans as ‘maintenance host’ (ref.^20^) entails interdependent roles of metabolism in mycobacterial cell physiology and mycobacterial pathogenicity. Second, since every stage of the *M. tuberculosis* life cycle — except for transient environmental exposure during transmission — occurs inside humans, often intracellularly, there is a reciprocal determinism such that host and bacillary function and behaviour are mutually and dynamically impacted and adjusted. The mycobacterial cell envelope exemplifies these principles, providing essential barrier functions and serving as the primary host–*M. tuberculosis* interface as site of immune recognition^81^, evasion, and modulation (Fig. 1)^71^. Its central role in *M. tuberculosis* biology is evident from the substantial genetic investment in the nanomachinery required for the construction, remodelling, and repair of this highly hydrophobic, multi-layered barrier that combines both Gram-positive and Gram-negative features, but is uniquely arranged with mycobacterial inner and outer membranes surrounded by an outer capsule (Fig. 1)^82^. The cell envelope of *M. tuberculosis*, possibly a neoderm^29^ that evolved de novo^83^, is not static, but instead has a structure and composition that are thought to change depending on the growth phase and the environment^84^. Key biophysical properties may impact host infection; for example, the hydrophobic lipid phthiocerol dimycocerosate (PDIM) adopts a conical shape that promotes phagocytosis of *M. tuberculosis* by macrophages^85^, while the capacity for morphotype switching may enhance intracellular survival^86^. The differential detectability of *M. tuberculosis* in clinical samples — including both viable but non-culturable and non-acid fast organisms^87^, as suggested by recent analyses of human ante- and post-mortem lung and lymph node samples^74^ — further implies the capacity of *M. tuberculosis* to adopt different morphotypes, potentially including ‘cell wall-deficient’ forms^88^. However, additional work is required to confirm their existence and role in human infection.

The identities, structures, and mechanisms of the systems enbling egress into, and import from the extracapsular environment of *M. tuberculosis* are also poorly understood, though several studies have expanded the catalogue of putative cell envelope proteins and their locations.^89^ How these systems are regulated remains unknown, especially considering the presumed need of the bacterium to evade immune detection in some environments and modulate immune function in others. The functions of the five T7SSs have been most thoroughly investigated, with studies implicating structural components and cargo in multiple pathways, including phagosome escape, inhibition of phagolysosome maturation, and recruitment of macrophages^90^. Consistent with their critical roles in modulating the host–mycobacterium interaction, these systems are among the T cell epitopes commonly detected in healthy individuals infected with *M. tuberculosis* as well as patients with TB^91^. The *Mycobacterium*-specific PE-PPE-family proteins are subject to secretion, and fulfil critical roles in nutrient acquisition^92^, and interference in host cellular and immune function^42^.

### Metabolic and physiological adaptation of *Mycobacterium tuberculosis* during tuberculosis

**Fig. 3:**
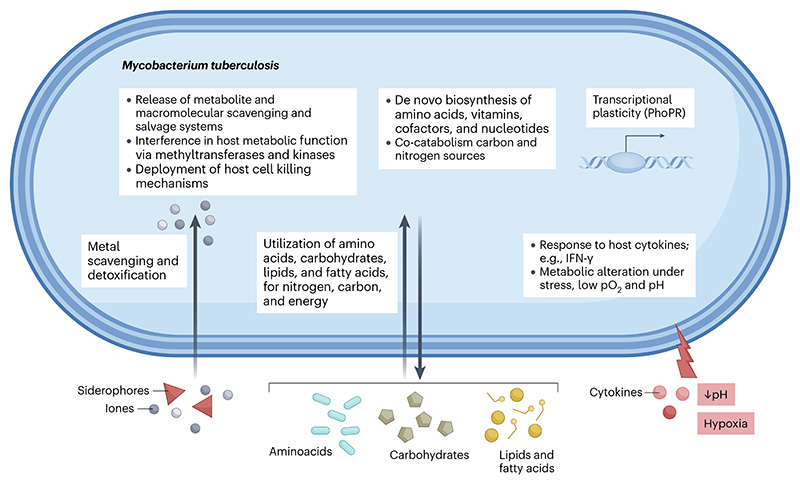
Metabolic capacity of *Mycobacterium tuberculosis* and metabolism during host infection. In silico, in vitro, intracellular, and animal models of *M. tuberculosis* infection have informed current knowledge of the metabolic repertoire of the bacillus^30^. *M. tuberculosis* down-shifts glycolysis and exploits host lipids for energy generation and biomass^194^, as well as acid resistance^195^. Fatty acids and cholesterol feed β-oxidation; carbon is channelled through the glyoxylate shunt, methylcitrate cycle, and B_12_-dependent methylmalonyl pathway to detoxify propionate while replenishing tricarboxylic acid cycle intermediates^46^. During hypoxia or nitric-oxide stress, a low-flux, highly reduced respiratory chain maintains ATP synthesis while lipids are stored for later use. *M. tuberculosis* also utilizes a range of nitrogen sources: host amino acids are deaminated, nitric oxide is detoxified, and the nitrate/nitrite reductase system supplies ammonia^93^. The bacillus scavenges inorganic phosphate, and assimilates sulfate for cysteine and mycothiol biosynthesis, supporting redox balance^96^. De novo vitamin B_2_, B_6_, and B_7_ pathways, plus cobamide (B_12_) salvage^107^secures essential cofactors. Dual-use virulence factors harvest transition metals while avoiding toxicity: mycobactin and carboxymycobactin siderophores sequester iron; zinc, copper and manganese ions support metallo-enzymes^104^. *M. tuberculosis* secretes protein kinases, and acid and tyrosine phosphatases, to block phagosome-lysosome fusion, sustaining a mildly acidic, nutrient-porous compartment^114^. Perforation of the phagosomal membrane enables cytosolic access to carbon and ions. Cell-envelope lipids and lipoarabinomannan dampen toll-like receptor signalling and arrest maturation, while proteases and antioxidant systems neutralize acid, reactive oxygen, nitrogen and chlorine species^110, 196^. Trehalose recycling conserves carbon while regulating envelope integrity. Under prolonged stress, the mycobacterial proteasome and the enduring hypoxic response redirect resources to repair and maintenance^197^, while toxin-antitoxin modules slow translation. Through this tightly integrated metabolic-virulence network, *M. tuberculosis* converts even hostile intracellular environments into privileged, nutrient-accessible refuges, enabling long-term intracellular persistence with the capacity for rapid re-initiation of replication.

A wealth of research has confirmed the unexpected prototrophy inferred from the *M. tuberculosis* genome, which encodes the capacity to biosynthesise most essential amino acids, vitamins, cofactors, and nucleotides, as well as utilise amino acids, carbohydrates, lipids (especially cholesterol), and fatty acids as sources of nitrogen, carbon, and energy during host infection. Additionally, the bacterium exhibits respiratory flexibility, enabling optimal growth in aerobic conditions and survival under hypoxia (Fig. 3)^30,46,93,94^. Efforts towards new TB drug discovery (Box 2) have advanced our understanding of gene and pathway essentialities and functions of *M. tuberculosis* in vitro and in different infection models, as well as elucidating the (macro)molecular compositions of different host environments (Box 1). These insights, coupled with advances enabled by TB vaccine research (Box 2) have enriched the understanding of how *M. tuberculosis* infection affects host immunometabolism and pathometabolism, while supporting the potential to generate multi-omic ‘atlases’ (ref.^95^) that describe the molecular and cellular characteristics of TB-defining lesions^7^. When combined with genomic insights^20,32^, a picture emerges suggesting metabolic and structural evolution from an ancestral environmental generalist, through a mammalian-adapted semi-environmental intermediate, to a bacterium equipped for human infection and transmission via aerosolization^82^.

Although in silico, in vitro, intracellular, and animal models have generated a catalogue of *M. tuberculosis* metabolic capacities (Fig. 3)^30^, the extent to which the identified pathways are relevant in human pulmonary and extrapulmonary TB (Fig. 1b) remains speculative. The metabolic flexibility of *M. tuberculosis* is amplified by its capacity for co-catabolism of carbon and nitrogen sources^96^, as well as modification of its response to different host cell types^97^. *M. tuberculosis* can adjust (and slow) its metabolism under stressful conditions^98^, including low oxygen tension^27^ and low pH^99^. The response to host environmental cues is enabled by a range of transcriptional regulators, among which PhoPR appears especially important in the evolution of *M. tuberculosis* for human infection^100^. Transcriptional plasticity facilitates stress adaptation^101^ through phenotypic diversity^33^. During infection, *M. tuberculosis* relies primarily on host-derived lipids as carbon sources for energy production and to synthesise its own complex lipids^46,93^. While *M. tuberculosis* can metabolize carbohydrates in vitro, gluconeogenesis predominates during infection^102^. The catabolism of cholesterol also produces acetyl-CoA, propionyl-CoA, and pyruvate, which are used in gluconeogenesis and the biosynthesis of cell envelope lipids. Pyruvate is also generated during incorporation of lactate, another carbon source abundant during infection, with recent evidence supporting the adaptation of *M. tuberculosis* to enhanced lactate metabolism^103^. This introduces an additional concept in the metabolic adaption of *M. tuberculosis* to infection, namely the dual requirement for utilization of host-derived metabolites while avoiding toxicity. Metals are good examples of this requirement: *M. tuberculosis* produces siderophores to scavenge iron and systems to detoxify excess copper within the macrophage^104^. Emerging evidence also indicates its capacity to monitor ion levels^105^. However, fundamental questions remain regarding the identities and specificities of the different transport systems enabling assimilation of nutrients, with recent evidence implicating putative ABC transporters in siderophore import^106^ and vitamin B_12_ acquisition^107^. There is growing interest in the application of chemical proteomics to understand host–bacterium interactions^108^, and to characterize the dual roles of transport systems in mycobacterial metabolism and interference in host cell signalling^42^.

### Metabolism at the host–bacterium interface

*M. tuberculosis* infection is often viewed from the perspective of the host withholding key nutrients and co-factors from the infecting bacillus, and the bacillus attempting to minimize the impact of this strategy through the deployment of host cytotoxic, scavenging, or salvage mechanisms^109^ or, in some cases, de novo biosynthesis. An example is the production of a mycobacterial cytotoxicity factor with NAD+ glycohydrolase activity, known as tuberculosis necrotizing toxin (TNT). This triggers the production of reactive oxygen species in *M. tuberculosis*-infected macrophages, which results in cell death and enables mycobacterial replication and access to cytosolic metabolites and cofactors^110^. In a rare demonstration of metabolite salvage, in vivo-adapted *M. tuberculosis* has been shown to salvage nicotinamide much more efficiently than cells grown aerobically in vitro^111^. On the other hand, the ability of *M. tuberculosis* to subvert host immunity was demonstrated in a screen that identified genes involved in tryptophan biosynthesis as key for bacillary survival against host CD4 T cell immunity, through the counteraction of tryptophan-depleting indoleamine 2, 3-dioxygenase (IDO) pathway^112^.

Less commonly considered is the extent to which *M. tuberculosis* directly interferes with host metabolic function via its array of methyltransferases and kinases. For example, there is some evidence that *M. tuberculosis* secretes the methyltransferase Rv2067c into macrophages, trimethylating histone H3K79 in a non-nucleosomal context^113^. The serine–threonine kinase PknG subverts the host innate immune response by modulating autophagy^114^, whereas PknF interferes with NOD-like receptor family pyrin domain-containing protein 3 (NLRP3) inflammasome activation^115^. A related mechanism involves *M. tuberculosis*-mediated inhibition of macrophage DNA repair, which promotes type-I interferon (IFN) pathway and scavenger receptor-A1 (SR-A1)-mediated lipid droplet formation^116^. There is also increasing evidence suggesting the capacity for *M. tuberculosis* metabolism to respond to signalling molecules such as cytokines, raising the potential that chemical communication between host and bacterium might be a key determinant of infection outcome^117,118^. It is important to note that the metabolic and replicative states of *M. tuberculosis* in specific microenvironments and/or in consequence of host immune detection or sequestration directly impact TB drug efficacy^94^, independently of drug distribution and penetration, which may be exacerbating the issue^119^ (Box 2). Thus, integral features of *M. tuberculosis* infection and immune survival present major barriers to chemotherapy, prolonging the duration of treatment and facilitating the acquisition of resistance.

### Microenvironments occupied by *Mycobacterium tuberculosi*s

The seemingly simple nature of the pulmonary infection cycle belies the difficulty in determining which metabolic and physiological factors are critical for *M. tuberculosis* to persist in humans. When the potential for extrapulmonary residence is factored into the problem, the scale of the challenge is amplified owing to the implied capacity of *M. tuberculosis* to occupy diverse host (micro)environments^120^. Comparative genomics of the MTBC has allowed some insights into the metabolic adaptations differentiating *M. tuberculosis* sensu stricto lineages from the animal-adapted ecotypes^20,32,37^. Additional resolution, especially of the functions under selective pressure across different regions and disease manifestations^121^, can be achieved when those comparisons are confined to *M. tuberculosis* alone. Nevertheless, the large metabolic repertoire of the bacterium remains subject to speculation. The apparent paradox — its autarky, or self-sufficiency, in a specialist pathogen — informs all studies of *M. tuberculosis* as the bacterial cause of TB. This difficulty arises from the common perception that *M. tuberculosis* is inherently virulent and pathogenic, which is limiting^122^. Given the long co-evolution of *M. tuberculosis* with humans^20,32^, this perspective appears overly simplistic.

The focus, too, on pulmonary disease, to the exclusion of all other extrapulmonary infections as terminal for onward transmission, might limit our appreciation of *M. tuberculosis* metabolism. As new tools have been applied, which offer greater sensitivity of detection, the list of potential cellular and anatomical niches has grown^77,88,123^. Occupation of specific environments in specialist structures might offer a mechanism to avoid host immune surveillance under some conditions, while stimulating recognition in others^71,124^. Does the conservation of T cell antigens^125^ with potentially stage-specific deployment during infection^91^ indicate a deliberate strategy by the bacterium to ensure initial transit across the lung epithelium and dissemination via cellular recruitment into granulomas? In this context, survivability is paramount, not stealth. If the broad metabolic, physiological and structural capacity of *M. tuberculosis* are required to occupy diverse microenvironments — including extrapulmonary sites — then the maintenance of a large genome necessarily implies that exit from those environments is under selective pressure. It seems improbable that all extrapulmonary infections are evolutionary dead ends. This conclusion is supported by the inferred association between phylogenetic lineage and site of disease in a recent study, which demonstrated a role for the T7SS effector, EsxM, in driving extrapulmonary dissemination through its effect on macrophage function^126^. If valid, the expansion of the natural lifecycle of *M. tuberculosis* to include extrapulmonary residence broadens the metabolic landscape under selective pressure, possibly resolving the paradox of large metabolic capacity in a human-adapted specialist.

## Host response to *Mycobacterium tuberculosis*

**Fig. 4:**
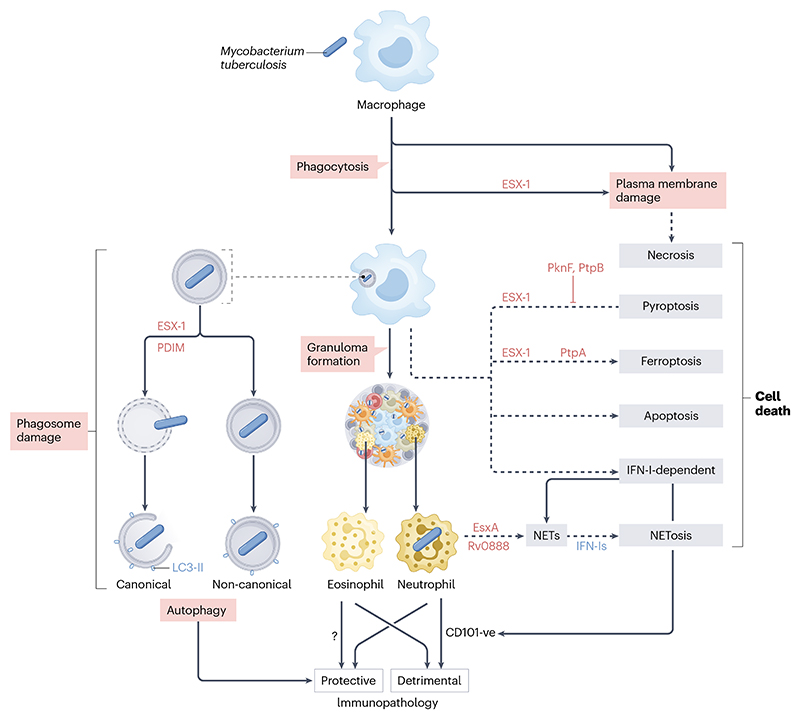
Host cell autonomous response to *Mycobacterium tuberculosis* infection. The *M. tuberculosis* factors implicated in the various response pathways are shown in brown, whereas host factors are shown in blue. During phagocytosis, *M. tuberculosis* can directly induce membrane damage mediated by the type VII secretion system, ESX-1. Plasma membrane damage can also occur following phagocytosis. In both cases, this leads to necrotic cell death^163^. Once in the phagosome, the activity of *M. tuberculosis* ESX-1 and the presence of *M. tuberculosis* lipids such as phthiocerol dimycocerosates (PDIMs) trigger damage of this organelle. This membrane damage triggers the lipidation of LC3 from LC3-1 to LC3-II as part of canonical and non-canonical autophagy responses that are primarily protective for the host. ESX-1-dependent activities have also been associated with other types of macrophage death including pyroptosis, ferroptosis and apoptosis^7,21^. A secreted *M. tuberculosis* effector, the phosphatase, PtpA, will induce ferroptosis^172^. *M. tuberculosis* can also block cell death pathways, and effectors such as the serine/threonine kinase, PknF, and the phosphatase, PtpB, can inhibit pyroptosis^115, 168^. ESX-1 activities can also trigger Type I interferon (IFN-I) responses from infected macrophages, the formation of granulomas, and the recruitment of other immune cells including eosinophils and neutrophils. Paradoxically, eosinophils seem to be protective, whereas neutrophils appear to be detrimental in the immunopathogenesis of tuberculosis. It is not known how eosinophils are protective (denoted by?), but in the case of neutrophils, IFN-I responses mediated by the *M. tuberculosis* proteins, EsxA and Rv0888, elicit the formation of neutrophil extracellular traps (NETs) which are associated with tuberculosis pathology^132^. IFN-Is also drive CD101-negative (CD101^-ve^) neutrophil recruitment to the lungs, which exacerbates tissue damage and pathology^198^. LC3, microtubule-associated protein 1A/1B-light chain 3; LC3-II, LC3-phosphatidylethanolamine conjugate.

In the following section, we provide an overview of certain mechanisms by which the host responds to *M. tuberculosis* infection. We focus specifically on the role of granulocytes in the immune response to *M. tuberculosis*; the mechanisms of damage and repair of the *M. tuberculosis*-containing phagosome; the role of autophagy in the innate immune response, and the functions of distinct cell death modalities in the context of *M. tuberculosis* infection (Fig. 4) before reviewing the spatial organisation of the TB granuloma. For further insights into TB immunopathogenesis, the reader is referred to several excellent reviews^7,71,127^.

### Granulocytes in the immune response to *Mycobacterium tuberculosis*

It is increasingly clear that myelocytic cells beyond macrophages are important during *M. tuberculosis* infection. Bronchoalveolar lavage (BAL) of patients with TB indicated that neutrophils are the main cells infected^128^ a finding confirmed in animal models. In mouse TB models, *M. tuberculosis* is preferentially found within long-lived cells in lesions^129^, which carry Ly6G, a cell surface marker for neutrophils, monocytes and granulocytes. Arguing for a beneficial role of neutrophils in *M. tuberculosis* infection, depletion of these cells in C3HeB/FeJ mice is detrimental to the control of mycobacterial replication by the host^130^. An inverse correlation has also been established between neutrophil numbers and the risk of TB in humans^131^. However, animal models suggest that neutrophils contribute to tissue damage and impaired bacterial control within lesions. Neutrophils recruited to the lungs of infected mice form neutrophil extracellular traps (NETs)^130^. In vitro, NET formation is mediated by the *M. tuberculosis* effector EsxA (also known as ESAT-6)^132^. The interplay between macrophages and neutrophils in granulomas is not fully understood, and the models used for their study may impact the precise relationship: Type I IFNs exacerbate disease in TB-susceptible C3HeB/FeJ mice by inducing neutrophil-mediated lung inflammation and NETosis (a cell death program characterized by the formation of NETs)^130^. In an alternate TB-susceptible mouse strain, NETs activate plasmacytoid dendritic cells to produce type I IFNs, which then blunt the interstitial macrophage response to IFN-γ^133^. Neutrophils likely also mediate *M. tuberculosis* transmissibility and access to new host cells and impact bacillary fate in permissive macrophages^134^. Consistent with findings from animal models, neutrophil abundance in blood and BAL correlate with TB severity in humans^135^. Thus, neutrophils both benefit and harm the host during *M. tuberculosis* infection.

Eosinophils have classically been linked to allergic responses and parasitic helminth infections. However, eosinophils are present in TB lesions in humans, resistant TB mouse models, and macaques^136^. In mice, eosinophil recruitment to the lungs is required for *M. tuberculosis* control and survival.^136^ However, it is unknown whether eosinophils found in human and macaque TB granulomas are protective or detrimental for disease^136^.

### Mechanisms of *Mycobacterium tuberculosis* phagosome damage and repair

**Fig. 5:**
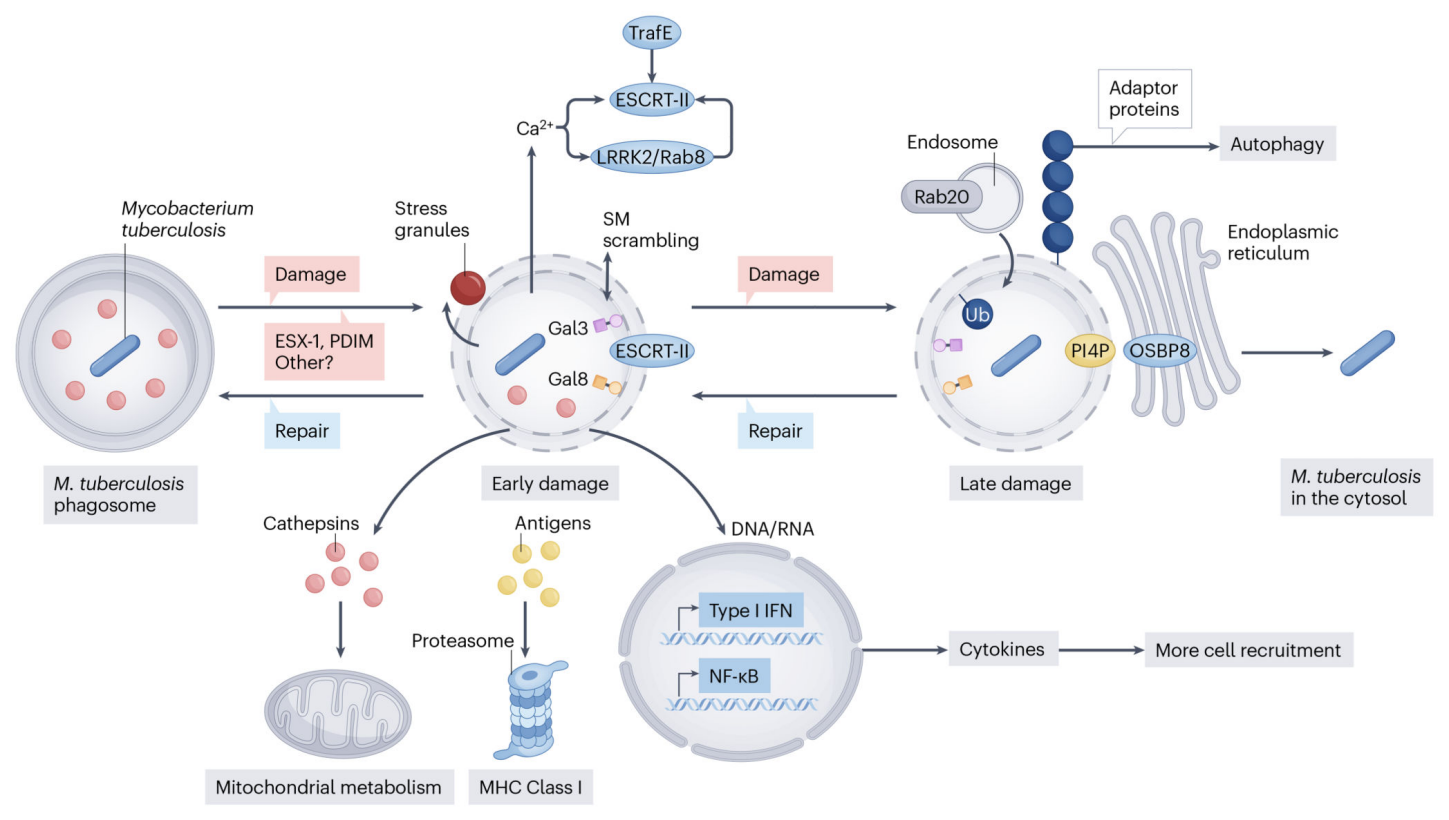
*Mycobacterium tuberculosis* phagosome damage and repair. In macrophages, *M. tuberculosis* is initially localised in a phagosome. Through bacterial components such as the type VII secretion system (T7SS), ESX-1, the cell wall lipid phthiocerol dimycocerosate (PDIM), and other unknown factors, the bacilli damage the membrane of the phagosome and access the cytosol. This membrane damage triggers mechanisms in the host cell, including the kinase, LRRK2 and its substrate, the Rab GTPase Rab8, the TRAF-like E3 ubiquitin ligase E (TrafE) and the ESCRT-III machinery^150^ which attempt to repair the damage. These pathways are triggered by signals from the lumen such as calcium (Ca^2+^) leakage^146^ and the scrambling of sphingomyelin (SM) caused by early damage of the membrane^143^. Membrane damage also triggers condensate formation and stress granule plug formation, which stabilises the membrane^140^. Cytosolic proteins such as Galectin-3 (Gal3) and Galectin-8 (Gal8) recognise carbohydrate moieties exposed from the lumen of the phagosome^146^. These sensors lead to the recruitment of adaptor proteins that in turn recruit repair factors including the autophagy machinery. If the early damage proceeds further, then other repair mechanisms are engaged. These include endosomal fusion with the phagosome mediated by Rab20^199^, a small GTPase that regulates phagosome maturation and trafficking; ubiquitination of damaged membranes, which involves the addition of a small protein, ubiquitin, to damaged lysosomal proteins, marking them for degradation; recruitment of the autophagy machinery through adaptor proteins; and the transfer of lipids from the endoplasmic reticulum through OSBP, a lipid transporter that facilitates lipid transfer between membranes at sites of contact, after accumulation of PI4P, a key lipid regulator of membrane signalling and trafficking, on the phagosome membrane^144^. If the damage continues and the phagosomes are not repaired, *M. tuberculosis* will translocate to the cytosol. During phagosome damage, bacillary and phagosome luminal components leak directly into the cytosol. These include antigens for cross presentation via major histocompatibility complex (MHC) Class I and lysosomal enzymes such as cathepsins that modulate mitochondrial metabolism by degrading specific mitochondrial proteins^151^. LRRK2, leucine-rich repeat kinase 2; ESCRT-III, endosomal sorting complex required for transport; Ub, ubiquitin; PI4P, phosphatidylinositol-4-phosphate; OSBP, oxysterol-binding protein.

The capacity to damage the phagosomal membrane is central to *M. tuberculosis* virulence, with key bacillary virulence factors such as the T7SS effector ESX-1 and the lipid PDIM contributing to this damage^137–139^. Increased attention has focused on understanding how the host responds to contain the damage (Fig. 5). Potentially among the earliest events in repair of *M. tuberculosis*-mediated phagosomal membrane damage, cytosolic stress granules form and ‘plug’ breaks in the phagosomal membrane, stabilizing the membranes until repair mechanisms are fully active^140^. The endosomal sorting complex required for transport (ESCRT)-III complex, which has been increasingly implicated in repair of damaged lysosomes, is recruited to the *M. tuberculosis*-containing phagosome in an ESX-1-dependent manner^141^. Notably, *Mycobacterium marinum* rapidly escapes to the cytosol in ESCRT-deficient *Dictyostelium discoideum*^142^, suggesting that ESCRT-III is also required to repair mycobacterial damage of the phagosomal membrane. Sphingomyelin scrambling and turnover precede ESCRT-III recruitment to lysosomes subjected to sterile damage^143^. While cytosolic sphingomyelin exposure has been identified as ESX-1-dependent in *M. marinum*-infected macrophages^143^, the full role of sphingomyelin remodelling in repairing *M. tuberculosis*-mediated damage has yet to be determined. Endoplasmic reticulum-dependent repair is a newly identified, ESCRT-independent mechanism for repairing damaged endomembranes. Repair-associated endoplasmic reticulum proteins are recruited to mycobacterial phagosomes in an ESX-1-dependent fashion, consistent with a role for endoplasmic reticulum-dependent repair of *M. tuberculosis*-driven damage^144^. As a fundamental understanding of endoplasmic reticulum-dependent repair is emerging, this area is fertile ground for future discovery. VPS18, a member of membrane trafficking complexes, was recently identified as an additional factor required for repairing the damage caused by *M. tuberculosis* to the phagosomal membrane^145^. Whereas the mechanisms of *M. tuberculosis* phagosome repair are becoming clear, less is known about the signals that trigger phagosome repair pathways. It is clear that one of the key signals is calcium. In this context, in addition to regulating ESCRT-III recruitment, calcium leakage-dependent lipidation of autophagy-related protein 8 (ATG8)/microtubule-associated protein 1A/1B-light chain 3 (LC3) limits phagosome damage caused by *M. tuberculosis* and restricts its replication^146^.

The role of autophagy (a form of cell death) in constraining damage to mycobacterial phagosomes is likely complementary to that of phagosomal repair mechanisms. *M. tuberculosis*-infected, induced pluripotent stem cell-derived macrophages that are unable to carry out canonical autophagy or non-canonical autophagy have defects in *M. tuberculosis* control. The mechanism is unknown, but possibly involves recapture of *M. tuberculosis* in the cytosol or direct repair of the phagosome^147^. Indirect evidence from the *Dictyostelium discoideum–M. marinum* model for cell-autonomous defence mechanism in response to mycobacterial infection^148^, and from *M. tuberculosis*-infected RAW264.7 cells, suggests that autophagy is triggered at sites of substantial membrane damage and bacterial exposure to the cytosol^142,149^. The LRRK2 kinase phosphorylates the small GTPase protein Rab8A to drive repair of *M. tuberculosis*-damaged phagosomal membranes, with deletion of LRRK2 resulting in a compensatory increase in autophagy^149^. The E3 ubiquitin ligase, TrafE, appears to be required for the induction of both ESCRT-III and autophagy upon membrane damage, as loss of TrafE in the *Dictyostelium–M. marinum* model causes loss of both processes and rapid host cell death^150^.

The essentiality of *M. tuberculosis* virulence factors that damage the phagosomal membrane supports the idea that such damage benefits the bacterium in its interaction with the host. However, the mechanisms by which that benefit accrues are incompletely known. Damaging the phagosomal membrane shifts cellular metabolism to reduce mitochondrial metabolism^151^, and changes the immune response. In *M. tuberculosis*-infected macrophages, phagosomal membrane damage drives a potentially detrimental type I IFN response^152^ and blunts a toll-like receptor 2 (TLR2)-dependent nuclear factor NF-kB response^153^. Additionally, damage to the phagosomal membrane modulates the adaptive immune response, enabling the presentation of *M. tuberculosis* antigens via major histocompatibility complex I (MHC-I) in infected cells, with consequent recognition by CD8 T cells^154,155^.

### Autophagy in response to *Mycobacterium tuberculosis* infection

As for many other intracellular pathogens, autophagy is part of the innate immune response of macrophages to *M. tuberculosis*. Autophagy-related (ATG) genes encode key components of the autophagy pathway, with knockout mice having enabled a growing understanding of the role of individual ATG genes in *M. tuberculosis* infection. Initial studies in resistant mouse models linked ATG5 function to inflammation^156^. Subsequent research showed that conditional knockout of ATG genes did not affect bacterial load in mouse strains that mimic chronic human disease, confirming only that ATG5 contributes to inflammation and neutrophil recruitment in vivo^157^. Studies using more advanced genetic models found that ATG5, ATG7 and ATG16L1 were key for control of *M. tuberculosis* in a resistant mouse model^158^. Loss of autophagy in lung macrophages and dendritic cells results in acute susceptibility of mice to high-dose *M. tuberculosis* infection, a model that reflects aspects of active human TB^159^. In this acute model, autophagy, instead of contributing to *M. tuberculosis* control in macrophages, dampens macrophage responses that drive the accumulation of myeloid-derived suppressor cells and subsequent defects in T cell responses^159^. In fact, ATG5 is required to suppress early T helper 17 responses in the lungs during *M. tuberculosis* infection^160^.

Despite these studies in TB-susceptible mice, the precise cellular mechanisms through which autophagy controls *M. tuberculosis* in macrophages ex vivo are incompletely understood. In part, this uncertainty arises from differences between mouse and human macrophages^147^. ATG proteins can have both canonical and noncanonical roles in cellular functions. In human macrophages, both ATG7 and ATG14 are required for the control of *M. tuberculosis*^147^. However, targeting to autophagosomes and, consequently, lysosomes does not completely explain restriction of *M. tuberculosis* by macrophages. Following *M. tuberculosis* damage of the phagosome membrane, either *M. tuberculosis* or host membranes are recognized by cytosolic sensors that target *M. tuberculosis* to autophagosomes^147^.

The overarching role of autophagy in controlling *M. tuberculosis* infection therefore remains a matter of debate. It is clear that factors required for canonical and non-canonical autophagy, such as ATG5, ATG7 and ATG16L1, reduce lung pathology in mice, but contradictory results raise questions about the degree to which they restrict *M. tuberculosis* replication in vitro and during acute and chronic infection in vivo^161^. The reasons for these discrepancies are numerous and likely include species differences (for example, mouse macrophages produce high levels of nitric oxide) and *M. tuberculosis* strain differences^161^. Studying the autophagy pathway in vivo is complex owing to the links between autophagy and stress responses as well as cell death. In this context, both conditional and full knockout mouse models are essential for analysis of the role of autophagy. However, core ATG gene-knockout mice show different phenotypes, but the underlying mechanisms are not fully elucidated. Compelling evidence shows that ATG proteins may have autophagy-independent functions, and some ATG proteins may not be essential even for canonical autophagy. These findings suggest that it might not be possible to determine the specific function of autophagy simply by deletion of a single ATG gene in mice^162^.

### *Mycobacterium tuberculosis* infection and host cell death pathways

As the spectrum of cell death modalities has been more comprehensively elucidated, understanding the roles that distinct modes play in the context of *M. tuberculosis* infection has become an area of interest (Fig. 4). Necrotic pathways of cell death are required for the formation of caseous granulomas, and subsequently for rupture into airways, with or without causing cavitation. Thus, *M. tuberculosis* requires necrosis to sustain transmission. Macrophages in cell culture offer the simplest model for studying *M. tuberculosis*-driven cell death but, even within this relatively homogeneous system, necrotic, apoptotic, and pyroptotic cell death occur simultaneously^163^. Growing evidence implicates mitochondrial health and function in cell death, including by pyroptosis^164–166^. NLRP3-driven pyroptosis, which is ESX-1 dependent^167^, is an active area of study. Surprisingly, it appears that the role of ESX-1 in pyroptosis is not through damage of the phagosomal membrane but, rather, damage to the plasma membrane^163^. Pyroptotic cell death is likely protective for the host, as the bacterium has evolved multiple mechanisms to interfere with pyroptosis. The *M. tuberculosis* serine–threonine kinase, PknF, interferes with NLRP3 inflammasome activation^115^, whereas the phosphotyrosine protein phosphatase, PtpB, interferes with NLRP3- or AIM2-triggered pyroptosis by serving as a phospholipid phosphatase, modifying plasma membrane phosphoinositols to interfere with Gasdermin D insertion at the plasma membrane^168^. Tools and interventions that enable the exploration of how pyroptosis might be harnessed to promote control of TB have yet to be fully developed.

A more recently described form of necrotic cell death, termed ferroptosis, is triggered by iron-dependent accumulation of peroxidated lipids, though the mechanistic details of the path to cell death are unknown. In a mouse macrophage model, *M. tuberculosis*-infected cells accumulate peroxidated lipids and die through ferroptosis^169^. Multiple pathways can reduce intracellular lipid peroxides to preserve cell health; in *M. tuberculosis*-infected macrophages, glutathione peroxidase 4 (GPX4) plays a critical role in blunting the accumulation of peroxidated lipids and reducing ferroptotic cell death^170^. The transcription factor BACH1 regulates the expression of antioxidant programs that impact induction of ferroptosis^171^. While most of the experimental work has been performed in mouse models, immunohistochemical studies on human lung tissue demonstrate that these molecular determinants of ferroptotic cell death are present around necrotic granulomas^170,171^. Small molecule inhibition of ferroptosis reduces tissue pathology and *M. tuberculosis* burden in mice^169^, suggesting that modulating ferroptosis could ultimately be used as part of a host-directed therapy to improve TB treatment outcomes. In a subsequent study, the *M. tuberculosis* phosphoprotein phosphatase PtpA was shown to serve as a secreted effector that triggers ferroptosis to promote pathogenicity in a mechanism independent of its phosphatase activity^172^.

The apoptosis of *M. tuberculosis*-infected macrophages has long been thought to contribute to host control of infection^173^. Investigating individual cell death modalities in vivo is complex given the multiple cell types present, varied states of infection, variety of cell death pathways induced by *M. tuberculosis* infection, and substantial pathway cross-talk. However, recent evidence from a mouse model demonstrates that apoptosis is host protective^174^. Apoptosis is induced by either an extrinsic pathway, triggered by a soluble factor binding a death receptor on the cell surface, or by an intrinsic pathway, triggered by caspase activation. Antagonists of inhibitor of apoptosis (IAP) proteins, which sensitize cells to activation of the extrinsic pathway, enhance apoptosis of *M. tuberculosis*-infected bone marrow derived macrophages and human monocyte-derived macrophages^174^. IAP protein inhibitors in a mouse model of TB infection modestly reduced bacterial burden and enhanced the number of ESAT-6-specific activated CD4 T cells^174^.

### Spatial organisation of the tuberculosis granuloma

The histopathologic hallmark of primary TB in humans is the granuloma, with individual *M. tuberculosis–*host cell interactions in granulomas potentially determining disease progression. Granuloma formation in human TB is highly heterogeneous and immune responses are spatially organized^175^. Recent advances in single-cell technologies have enabled a richer, more nuanced understanding of the composition and cellular phenotypes of lung-associated lesions in humans and mouse models than was previously possible (Box 1). Single-cell RNA sequencing (scRNA-seq) applied to profile the lungs of mice infected with a fitness fluorescent reporter strain of *M. tuberculosis* identified subpopulations of pro-inflammatory alveolar macrophages infected with stressed bacilli and interstitial macrophages infected with *M. tuberculosis* across a range of physiologic states^176^. Supporting the clinical relevance of these findings, similar macrophage subpopulations were identified in BAL fluid from patients with TB. These macrophage populations in the lung are epigenetically constrained in their response to infection^176^.

ScRNA-seq has captured important responses at the single-cell level and has enabled the identification of unique cellular subpopulations. Seq-Well, a low-cost platform for massively parallel scRNA-seq, provided an early glimpse into the potential of this technology when applied to profile thousands of primary human macrophages exposed to *M. tuberculosis*^177^. By identifying distinct cellular clusters that displayed unique stimulation-induced shifts in the expression of genes associated with cell growth, hypoxia or metabolism, this analysis revealed how the response to *M. tuberculosis* can be influenced by basal host cell heterogeneity^177^. scRNA-seq additionally enabled the identification of the cells producing IFN-β in mice and in non-human primates during chronic infection^133^. However, on its own, scRNA-seq lacks spatial resolution. The development of spatial transcriptomic and proteomic approaches has allowed the integration of molecular and spatial information in human disease by linking transcriptomes at the single-cell level with spatial information at the tissue level. In mice, spatial and temporal localization of immune transcripts defines hallmarks and diversity within the individual TB granuloma. Encapsulated granulomas from C3HeB/FeJ mice (a valuable model that recapitulates key features of the immunopathology of human TB) show necrotic cores with transcripts primarily associated with immunosuppression (*Foxp3, Il10*), whereas those in the granuloma rims associate with activated T cells and macrophages^178^. Multimodal profiling of phenotypically distinct lung granulomas in macaques shows cellular correlates of bacteriologic control^179^. Bacterial persistence occurred in granulomas enriched for mast cells, endothelial cells, fibroblasts, and plasma cells with a strong type 2 immunity and wound-healing signature. Granulomas associated with the control of *M. tuberculosis* contained cellular populations enriched for type 1-type 17, stem-like, and cytotoxic T cells engaged in pro-inflammatory signaling^179^.

The application of multiplexed ion beam imaging by time-of-flight (MIBI-TOF) to image 37 proteins in individual immune cells within lung lesions from patients with active TB similarly demonstrated spatial organisation of immune responses within granulomas^180^. Highly localized immune modulation, such as IDO1- and PD-L1-expressing myeloid cells, proliferative regulatory T cells, and high levels of transforming growth factor (TGF)-β were noted alongside depletion of IFN-γ. IDO1-expressing myeloid cells appeared specific to TB granulomas, whereas PD-L1-expressing myeloid cells and a scarcity of activated T cells were also found in sarcoidosis-associated granulomas. Lastly, in an orthogonal analysis of blood transcriptomes from patients with TB, similar immunoregulatory expression dynamics defined systemic immunity during active disease^180^. In complementary work, multiplex Opal immunofluorescence was used for high-resolution mapping of the tissue-wide immune landscape in 13 patients who underwent partial lung resection following failure of antibiotic therapy. In addition to necrotizing granulomas, the human lung with TB was noted to contain abundant non-necrotizing leukocyte aggregates surrounding areas of necrotizing tissue^181^. These cellular lesions are more diverse in cellular composition than necrotizing lesions based on the spatial distribution of B cells and macrophages. The cellular composition of non-necrotizing structures also correlates with their proximity to necrotizing lesions, suggesting foci of distinct immune reactions adjacent to necrotizing granulomas. Therefore, during *M. tuberculosis* infection, lung tissue develops a histopathological superstructure of four different types of non-necrotizing cellular aggregates organized as satellites of necrotizing granulomas^181^.

## Conclusions and outlook

As outlined in this Review, the *M. tuberculosis*–host interaction is characterised by a complex and multifaceted set of counteracting responses, which ultimately determine infection outcome. By providing critical insights into the most recent evidence, we have attempted to highlight knowledge gaps. Here, we elaborate on these and propose questions that we believe should be addressed to better inform intervention strategies.

Outside TB drug discovery programs, which tend to prioritise target essentiality and vulnerability (Box 2), most studies of *M. tuberculosis* metabolism have been focused on identifying the repertoire of metabolic pathways available to the microorganism, providing limited understanding of their relevance during host infection. Nevertheless, studies performed to date support the conclusion that total ‘metabolic capacity’ might be the major determinant of *M. tuberculosis* virulence^22,23,182^. According to this hypothesis, which prioritises host occupation above virulence, disease is an incidental outcome that manifests in only a fraction (albeit enormous in absolute numbers) of individuals infected with *M. tuberculosis*. This notion is consistent with the contention that pathogenicity is not an intrinsic property, but instead an emergent outcome of a specific host–bacterium interaction^183^. In TB-endemic regions, prevailing environmental and socioeconomic factors^35^ combined with frequency of *M. tuberculosis* exposure increase the likelihood of a pathogenic interaction (that is, disease) owing to their impact on population health, immune function, and inflammatory status.

Does this exclude a role for *M. tuberculosis* in actively influencing the outcome of infection? Almost certainly not. The idea that *M. tuberculosis* passively resides in the host, infecting a large number of individuals without causing clinical symptoms while causing disease in a fraction, cannot be fully reconciled with evidence of *M. tuberculosis* virulence^184^, including its capacity to modulate and even provoke host responses^71^ that promote pathology and accelerate transmission. Future research should investigate how *M. tuberculosis* ‘monitors’ the condition of the host and identify the mycobacterial factors that trigger immunopathology, facilitating its exit and transmission to a new host when deteriorating host health (or any other disruption of the host-mycobacterium interaction) poses a risk to the bacillus reaching terminal occupancy. For now, we propose separating *M. tuberculosis* metabolism from the limiting constraints of virulence and reframing the prevailing paradigm of the host–*M. tuberculosis* interaction to accommodate a broader conceptualisation of host occupancy, and transmission, in the absence of immune elimination and host pathology. This argument, in turn, raises important questions about how well animal and other models reflect individual pathogenic aspects of *M. tuberculosis* infection in humans. Alternative systems are being developed to study cellular and immune responses in TB^77,88^. Further work is required to demonstrate their utility in dissecting the role of homeostatic and innate pathways, such as autophagy, in human disease. How individual cell death modalities intersect and contribute to complex tissue phenotypes and whether as-yet unidentified cell death modalities^185^ are at play in TB pathogenesis are additional unanswered questions. The use of high-content analyses to elucidate spatial localization and cellular compositions in hallmark pathological features of TB offers great promise^180,181,186^. At a mechanistic level, it is important to understand if and how the earliest events during infection — including *M. tuberculosis*-induced membrane damage in the first infected cells — define the spectrum of cell-intrinsic and local responses that dictate the outcomes of individual infection events. Ultimately, it remains to be determined whether a deeper understanding of the heterogeneous nature of *M. tuberculosis* infection can be used to focus therapies on molecular interactions or subpopulations of cells most critical for driving disease outcomes.

## Box 1 Models to study the biology of *Mycobacterium tuberculosis*

### Axenic culture

The standard conditions for phenotyping *Mycobacterium tuberculosis* strains entail aerobic culture of replicating bacilli in Middlebrook 7H9 media. In vitro models of non- or slowly-growing *M. tuberculosis* utilize lipid carbon sources, and combinations of hypoxic, nitrosative, acidic and/or nutritional stresses^200^.

### In vitro granuloma, organ-on-a-chip and human tissue models

Various cell culture systems of tuberculosis granulomas have been developed and applied in studying pathogenesis^78,201^. Human airway organoids show promise for deciphering early steps in infection^79^. A murine lung-on-a-chip model was used to investigate the role of pulmonary surfactant in early infection, and showed that alveolar epithelial cells play an important role in controlling *M. tuberculosis* growth^202^. It was also used to demonstrate that *M. tuberculosis* adapts to the alveolar microenvironment by forming cords^77^.

### Mouse models

In addition to the C3HeB/FeJ mouse, which presents with caseous necrotic pulmonary lesions reminiscent of human tuberculosis (and is applied in drug testing^203^), collaborative cross mice, used in conjunction with a transposon mutant library of *M. tuberculosis*, have been leveraged to identify host–pathogen interactions underlying tuberculosis susceptibility^182^.

### Non-human primates

The cynomolgus macaque and marmoset models, which recapitulate the spectrum of human tuberculosis and develop the full range of granuloma types, have had a transformative impact, especially when applied with positron emission tomography (PET) and computed tomography imaging^204–207^.

### Imaging and single-cell approaches

New PET probes of *M. tuberculosis* metabolism, such as 2-[^18^F]fluoro-2-deoxytrehalose, which allow the specific imaging of tuberculosis lesions, offer exciting opportunities for future studies^207,208^. Single-cell approaches are also impacting on the understanding of the immunopathogenesis of tuberculosis in humans and in animal models through the analysis of samples from host cells and bacilli coming from lung biopsies, bronchoalveolar lavage, pleural fluids, blood and bioaerosol^176,209^. Single-cell sequencing and analyses allow host cell type identification at the single cell level and the identification of disease phenotypes. Tissues can be analysed by spatial imaging with single-cell RNA spatial transcriptomics, multiplexed ion beam imaging by time-of-flight or multiplex labelling to identify specific cell populations and single-cell environments in tissues associated with markers of tuberculosis progression and granuloma formation. These studies are contributing to our understanding of single-cell responses in tissues (see text). In parallel, new methods have been developed to capture, detect and quantify viable aerosolized *M. tuberculosis* and applied to demonstrate aerosolization of *M. tuberculosis* by tidal beathing, and to show that aerosolization, undetectable by standard tests, is common in a highly tuberculosis-burdened area^52^.

## Box 2 New therapeutic and preventive strategies for tuberculosis

A tuberculosis drug pipeline has been established through cooperation between the public, private and academic sectors^210^. Advances in understanding the pathology along the spectrum of disease states^192^ and the implications for the variable environments encountered by *M. tuberculosis*^211^ have impacted the discovery of new tuberculosis drugs^212^. Phenotypic heterogeneity is a key survival strategy of *M. tuberculosis*^17,26^, which affects the organism’s intrinsic drug susceptibility. This complicates drug discovery by prioritizing the identification of compounds that not only disrupt cellular processes essential for the survival of *M. tuberculosis* in a state of non- or slowly-replicating persistence in which it displays phenotypic drug tolerance^213^, but are also able to access the organism in the complex host microenvironments^214^. Caseum has emerged as a niche of particular interest^80,215^. This has led to the establishment of a surrogate caseum model to screen for compounds that can kill non-replicating *M. tuberculosis*^80^. In parallel, insights from gene vulnerabilities in *M. tuberculosis*^216^ offer the prospect of prosecuting new highly vulnerable targets^217^. Approaches to designing treatment-shortening regimens that effect durable cure is another key area of research. Methods to systematically measure high-order drug interactions in *M. tuberculosis* provide a means of prioritizing drug combinations for pre-clinical evaluation in more costly models such as the relapsing mouse model^218,219^.There is also substantial interest in developing host-directed therapies for tuberculosis, an area underpinned by advances in understanding host–pathogen interactions, which can guide approaches to enhance immunity or ameliorate immunopathology. This topic has been covered in recent reviews^71,220,221^.

Tuberculosis vaccine research and development has been energized by promising results from clinical trials^222^ and by evidence of high, and even sterilizing, protection against tuberculosis by intravenous administration of Bacillus Calmette-Guérin (BCG)^205^ and other administration protocols in non-human primates^223^. Together with advances in tuberculosis immunology relating to the development of protective immunity^127^, these studies have provided a rich resource for discovering immune correlates of vaccine-induced protection against tuberculosis^224^. The 49% protection from tuberculosis observed in individuals with prior *M. tuberculosis* infection by the M72/AS01_E_ (ref.^222^) vaccine comprising a recombinant fusion protein, M72, derived from two immunogenic *M. tuberculosis* antigens, PepA (Mtb32A) and PPE18 (Mtb39A), and the adjuvant, ASO1_E_, has inspired a phase 3 clinical trial that is enrolling in sites across seven countries. In an important advance towards the rational selection of *M. tuberculosis* antigens for candidate subunit vaccines, profiling the T cell receptor repertoires to *M. tuberculosis* in infected individuals who either progressed to tuberculosis disease or controlled the infection, identified EsxB (CFP10), PE13, Wbbl1, and PPE18, as prime candidates by virtue of their recognition by mycobacteria-specific T cells associated with control of infection^209^.
